# Microscope-assisted, rodent whole heart perfusion fixation for histological characterization of myocardium remodeling

**DOI:** 10.1016/j.mex.2025.103417

**Published:** 2025-06-03

**Authors:** Cary Karcher, Anthony Campbell, Dov Levine, Morgan Moroi, Yaagnik Kosuri, Arianna Adamo, Giovanni Ferrari

**Affiliations:** Department of Surgery, Columbia University; New York, NY 10032, United States

**Keywords:** Perfusion, Fixation, Cardiac Remodeling, Myocardium, Rodent, Histology, Microsurgery, Microscope-Assisted Rodent Whole Heart Perfusion Fixation

## Abstract

Investigating mechanisms of heart disease, its progression, and developing therapeutics that target cardiac remodeling often requires translational model organisms. *In vivo,* rodent models have been well established as a means to explore heart pathologies for their physiologic similarities, low cost, reproducibility, and breeding efficiency. Chemical fixation and harvest of cardiovascular structures is an essential preliminary step to histological assessment that enables the characterization of macro and subtle tissue changes. Our method establishes a novel and reproducible strategy for optimal cardiac perfusion, cardiectomy, and chemical fixation of the whole rodent heart. Our non-survival, microscope-assisted surgical technique allows for robust and efficient fixation of the rodent myocardium, heart valves, and vasculature preserving precious features in preparatory steps for histological staining.

• Optimized method to sample blood, exsanguinate and thoroughly perfuse fixative through the left and right side of the whole rodent heart.
• Protocol is atraumatic to the ventricles and surrounding structures; Injections are into the inferior vena cava (IVC) and left atrium.
• Performed using accessible microsurgical tools and aided by a dissecting light microscope.

This novel method optimizes the preservation of rodent myocardium for histological study without the drawbacks of the current standard technique, which punctures the myocardium and inadequately evacuates blood products from the heart and vessels.

## Graphical abstract

**Figure:**
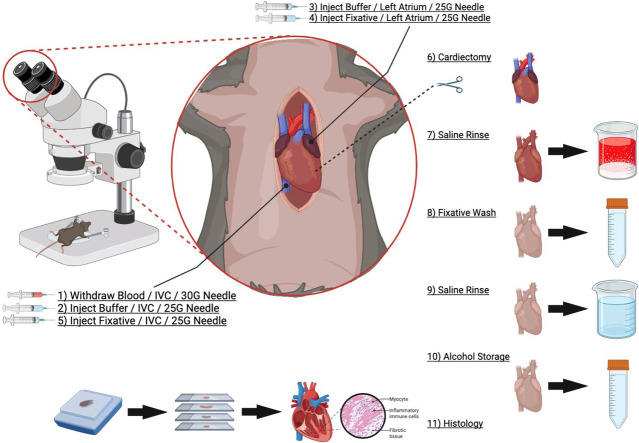

Specifications table

**Table:** 

Subject area:	Medicine and Dentistry
More specific subject area:	Heart Tissue Fixation
Name of your method:	Microscope-Assisted Rodent Whole Heart Perfusion Fixation
Name and reference of original method:	Gage GJ, Kipke DR, Shain W. Whole animal perfusion fixation for rodents. J Vis Exp. 2012 Jul 30;(65):3564. doi:10.3791/3564. PMID: 22871843; PMCID: PMC3476408.
Resource availability:	LEICA S9i Digital Stereo Microscope: https://www.leica-microsystems.com/products/light-microscopes/stereo-microscopes/p/leica-s9-e/

## Background

Cardiovascular disease (CVD) remains the number one cause of death globally, with approximately 20 million deaths reported in 2022 [1]. CVD results in more deaths than cancer and accidental deaths combined, the second and third largest killers [2]. The incidence of morbidity and mortality from heart disease continues to rise in both low income countries and high income countries [3]. Therefore, researching the pathogenesis and progression of CVD remains of significant interest to address this alarming problem. The development of small mammal models to assist in this investigation has increased in utility.

Rodent models of heart valve disease, coronary disease and heart failure have become increasingly popular for their translational nature [4]. One area of interest is the progression of cardiac remodeling and myocardium fibrosis as a driver of heart failure. The manner in which cell signaling abnormalities and alterations to mechanical transduction drive myocardial remodeling remains incompletely understood. Therefore, a reproducible method that allows for the robust fixation of living myocardium and subsequent histological examination of harvested tissue is required.

**Fig. 1:**
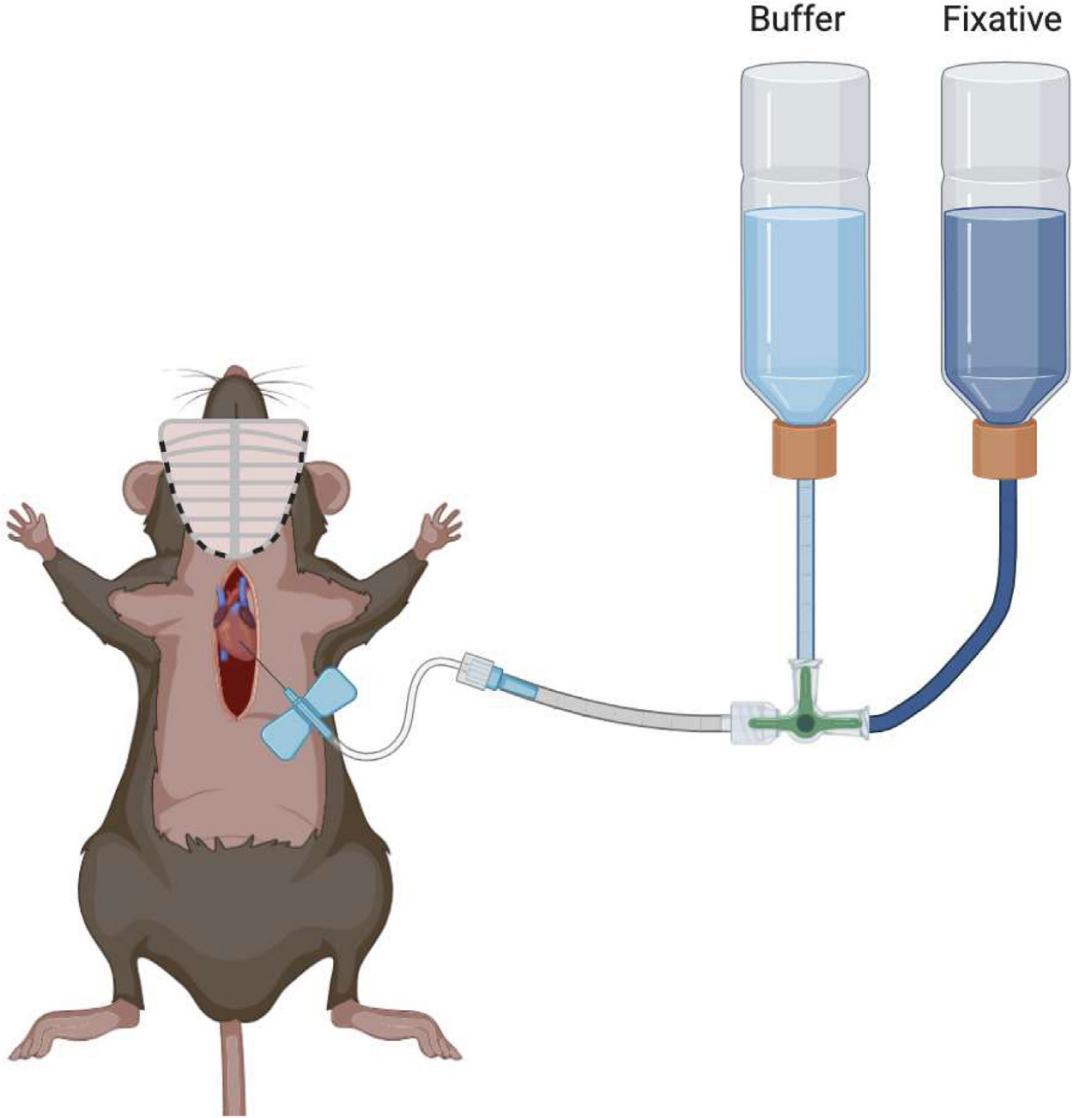
Standard perfusion fixation surgery technique.

The presently used standard perfusion fixation strategy results in an unwanted insult to the left ventricle and apex regions of the myocardium [5]. This results from inserting a needle into the left ventricle through the myocardium for administration of a buffer and a cross-linking fixation solution. As a result, penetrating injuries can be appreciated on subsequent histological sections, potentially complicating the intended analyses. The standard technique also commonly leaves incompletely flushed blood within the heart chambers, which form large clots surrounding heart valves and in coronary vessels. The clots create undesirable artifacts that compromise histological assessment of tissue samples. In this updated method, we establish a novel strategy for whole rodent heart perfusion fixation that preserves myocardial and valve integrity while achieving a more complete blood evacuation, further optimizing specimens for histochemical analysis. This method can be adapted for both mice and rats; for the purpose of this manuscript, we will focus on the mouse protocol exclusively (Fig. 1).

## Method details

The mice used for this method are part of a larger study being undertaken at Columbia University Irving Medical Center. This study seeks to investigate the progression of myocardial fibrosis and valvular remodeling as a result of dysregulations in serotonin signaling. Wild-type C57BL/6 J mice (male and female) and transgenic mice with knock-out serotonin transporter (SERT) B6.129(Cg)-Slc6a4 (male and female) were used to develop and evaluate this protocol. This technique was designed to ensure thorough fixation of intact whole mouse hearts while minimizing myocardial and valvular damage caused by apical needle insertion and insufficient blood clearance. All personnel, procedure locations, administered drugs, reagents, equipment, surgical procedures, and endpoints should be well-defined and included within the IACUC protocol.

### Materials

The microscope used for our dissection is the LEICA S9i Digital Stereo Microscope using 0.61x zoom and 10x eyepiece magnification for a total of 6.1x magnification. The scope is fitted with dual brightlight attachment to reduce shadow and improve surgical visualization. Other makes and models may also be appropriate. A rodent anesthesia system is required for this non-survival procedure. The anesthesia cart should be equipped with pressurized oxygen tank attachment (95% O2, 5% CO2), isoflurane vaporizer and gas mixing component. The gas collection waste chamber, isoflurane level, pressure gauge and carbogen gas volume should be checked and accurate. The anesthesia cart should contain adequate dual-flow tubing, a Y connector hose with 2-directional stop cock, air-tight induction chamber and nose cone. A glass petri dish is used to secure the mouse on the working stage throughout the surgical procedure, with heating pads and adjuncts.

Micro-dissection and standard surgical tools were used for tissue harvest. The sterile tools recommended are straight and bent dissecting microforceps, straight and bent grasping microforceps, ophthalmic microscissors, rat tooth forceps, hemostat, and curved scissors. 30 Gauge (G) needles and 1 mL insulin syringes were used for phlebotomy. A 25 G needle was used for the left atrial and inferior vena cava punctures and the administration of buffer and fixative agents. A 10 mL syringe was used for buffer injection and a 20 mL syringe for injection of the fixative. Nair, cotton swabs, and sterile saline were used to remove fur at the incision site. Blood collection and storage will be accomplished by 1 mL MiniCollect K3EDTA anticoagulation vacuum-sealed tube.

Ophthalmic ointment (Dechra-Ophthavet) is applied preoperatively to prevent drying of the membranes in the eye. Heating pads should be warmed to 35-38°C and placed under the supine mice. PPE such as a mask, head cap, surgical gown, and non-sterile gloves is recommended during surgical steps; sterility is not required for this terminal procedure. An antiseptic bactericide solution should be applied using cue tip to cover the surgical site pre-operatively, 7.5-10% povidone-iodine solution.

### Reagents and solutions

The buffer used for exsanguination is 1x PBS stored at 4°C. A mouse requires approximately 4-6 mL of buffer for every 25 g of body weight for blood clearance [6]. The fixative used for cross-linking tissue is 4% paraformaldehyde (PFA) in 1x PBS. The appropriate dilution of 4% PFA can be purchased from Thermo Fisher (CAS Number 30525-89-4). 4% paraformaldehyde can be stored at room temperature, 25°C but should be cooled to 4°C before injection. Approximately 15-20 mL is needed for 25 g body weight [7]. For anesthesia, we use 99.9% isoflurane (Covetrus) dosed 3-3.5% by inhalation mixed with carbogen gas 95% O2, 5% CO2. Sterile saline (NaCl) at 4°C is used for washing the harvested organ following cardiectomy. Falcon tubes with 5 mL 4% PFA at 4°C should be prepared prior to tissue removal. Falcon tubes with 5-7 mL of 70% ethanol at room temperature should be prepared for the post-fixative storage period. 100 mL beaker with ice-cold NaCl was used for rinsing steps.

Protocol:

Step 1: Mouse anesthesia and preoperative preparation

1. Before any animals are brought to the procedure suite:
  a. Gather appropriate sterile surgical tools (straight and bent blunt/dissecting micro forceps, ophthalmic scissors, rat tooth forceps, sharp-tipped Metzenbaum scissors, hemostats, cue tips).
  b. Aliquot wash solutions (50 mL NaCl 4°C, 5 mL 4% PFA 4°C, 5-7 mL of 70% ethanol 20°C).
  c. Prepare syringes and injectable solutions
    i. (30 G + 1 mL syringe - empty)
    ii. (25 G + 10 mL syringe - 4-6 mL 1xPBS 4°C)
    iii. (25 G + 20 mL syringe - 15-20 mL 4% PFA 4°C)
  d. Label tissue and blood collection containers for mice ID, age, sex, genotype, and date.
  e. Assemble petri dish, tape restraints, connect microscope and lights, fill and test anesthesia system.
  f. Gather PPE (Head covering, facemask, gown, gloves) and bactericidal solution.
  g. Check expiration dates for isoflurane, gas waste collection container weight and anesthesia systems, confirm equipment cleared for use.
2. Load mouse into anesthesia induction chamber and secure lid with oxygen flowing at 3-5 L. Adjust isoflurane from “off” to 3.5% once the animal is inside the chamber. Ensure that gas is being directed into the induction chamber and NOT the nose cone (Fig. 2 - Step 1).

**Fig. 2:**
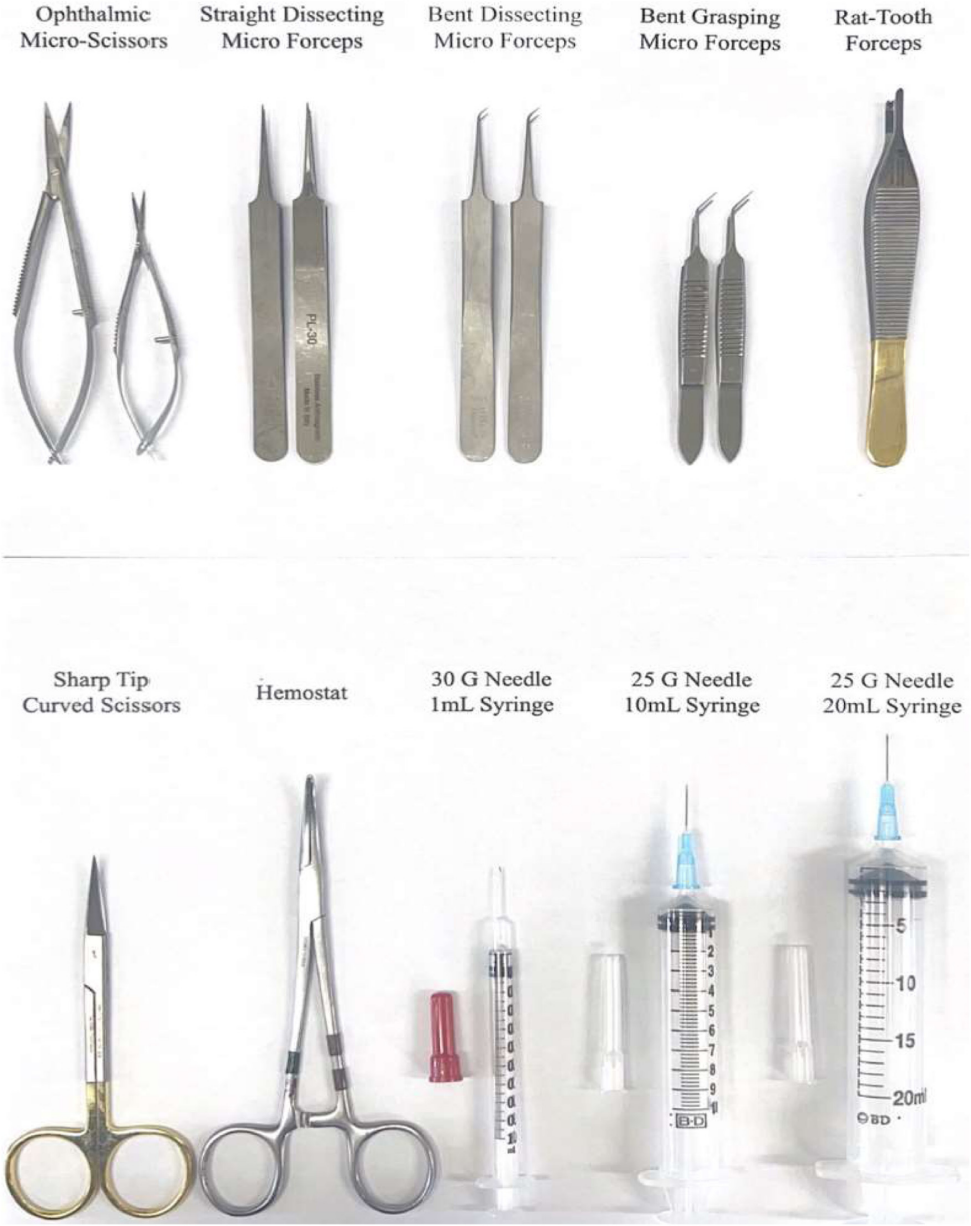
Perfusion surgery instruments.
3. Wait 3-4 minutes or until the mouse has been visibly asleep for 1 minute.
4. Transfer the mouse from the induction chamber to a large glass petri dish positioned in the center of the microscope stage. Place the mouse in the supine position on a hand-warmer with nose and jaw snug inside the nose cone (Fig. 2 - Step 2).
5. Decrease the isoflurane to 3.0%, and use tape to immobilize both the tail to the base of the stage and the mouse’s chin/head to the nose cone. Ensure the chest is centered in view of the microscope eyepiece and fix the overhead lights on the surgical site.
6. Use a cue tip to cover the mouse chest and upper abdomen with NAIR. Let the NAIR sit for 1-2 minutes, wipe clean with a warm, damp paper towel to remove NAIR and fur (Fig. 2 - Step 3).
7. Generously apply Dechra-Ophthavet ophthalmic ointment to mice eyes and manually open and close eyelids to distribute ointment (Fig. 2 - Step 3).
8. Using either 70% ethanol or 7.5-10% povidone-iodine solution, use a cue tip to apply the bactericidal solution around the incision site. Start in the center and work outwards, not returning to a location that has previously been scrubbed. Do this 3 times (Fig. 2 - Step 3).
9. Monitor the movement of the chest to ensure deep, steady breathing.
10. Using a rat tooth forceps, pinch both feet to confirm the surgical plane of anesthesia (Fig. 2 - Step 4).
  a. If leg withdrawal: wait or increase isoflurane
  b. If no reaction: continue the procedure

Step 2: Perfusion surgery: heart exposure

*This section****DOES NOT****require utilization of the dissecting microscope*.

1. Using rat tooth forceps, grasp the skin below the xiphoid process. Use medium, sharp tip curved scissors, to incise the skin parallel to the sternum to expose the underlying abdominal musculature.
2. Use rat tooth forceps to lift the abdominal wall and use the scissors to shallowly incise into and through the abdominal wall laterally, taking care to avoid any organs or major vessels (Fig. 3 - Step 1).

**Fig. 3:**
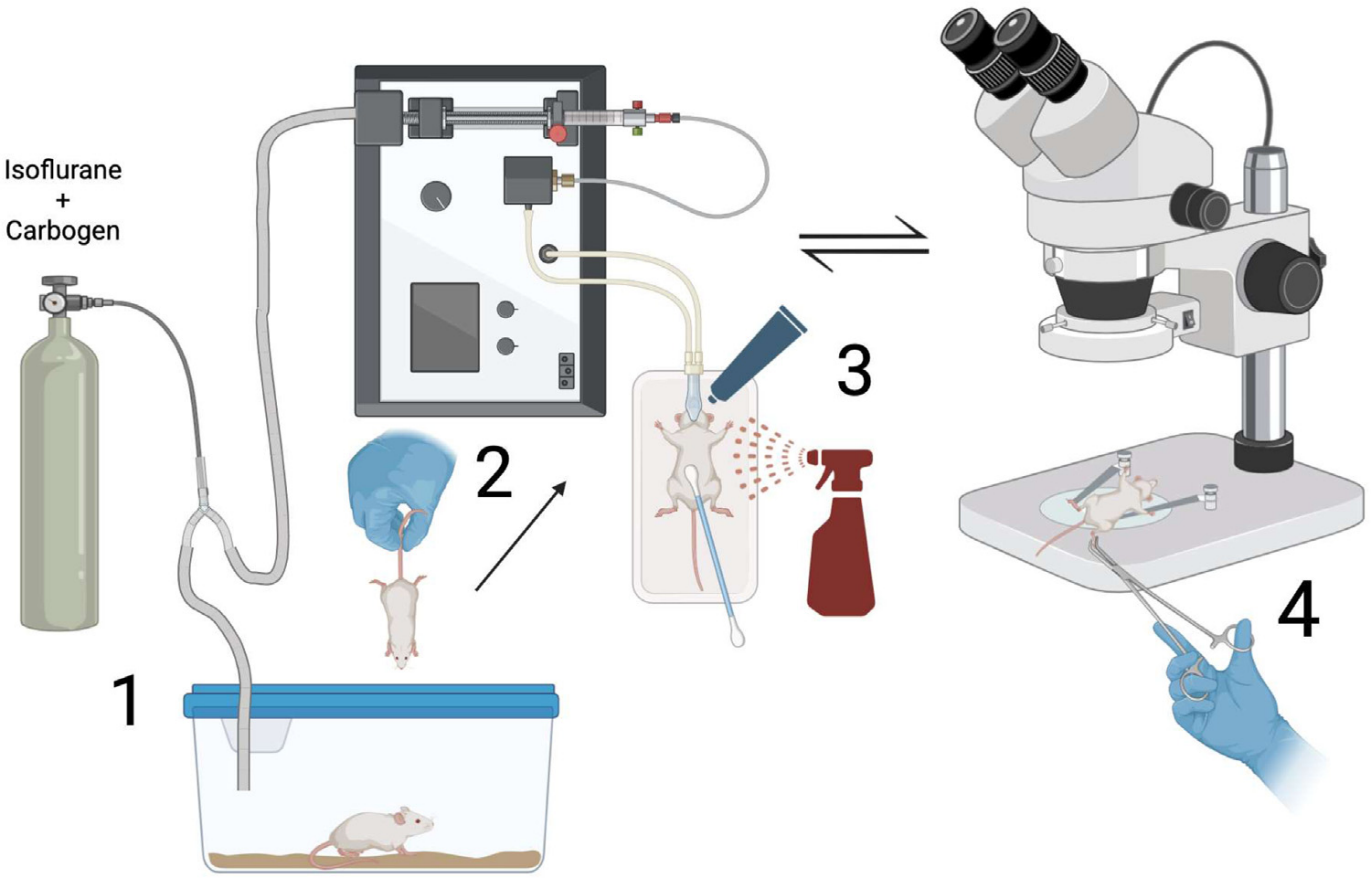
Mouse anesthesia and microscope-assisted surgery preparation. Confirm **BETH**: **B**reathing rate, **E**ye ointment, **T**oe pinch plane of anesthesia, and **H**eating pad.
3. Alternating sides, carefully insert the tip of the scissors into the abdominal cavity to extend the incisions laterally. Maintain visualization of the tip of the scissors to prevent inadvertent injuries to surrounding structures.
4. Transect the falciform ligament, to assist mobilizing the liver down and out of the surgical field.
5. Extend the lateral incisions cranially toward the diaphragm, so that the abdomen opens in a “U” shape (Fig. 3 - Step 2).
6. At the subcostal margin, sharply dissect the diaphragm from its thoracoabdominal wall attachments, fully connecting the abdominal and thoracic cavities. Be careful to avoid injuring the liver, lungs, heart, and major vessels (Fig. 3 - Step 3).
7. Transect the rib cage along the mid-axillary lines in the cranial direction bilaterally to allow its elevation (Fig. 3 - Step 4).
8. Use a hemostat to clamp down on the xiphoid process with the instrument oriented perpendicular to the ribs. Roll wrist forward to fold the “U” shaped flap section of thoracoabdominal wall and skin back over the face of the mouse exposing the mediastinum and thoracic cavity (Fig. 3 - Step 5).
  *Note: Ensure that the positioning of the mouse nose has NOT disengaged from the anesthesia cone when folding this flap*.
9. Create a posterolateral thoracotomy at the level of the 5th-8th ribs to facilitate drainage of the effluent from the thorax during perfusion, thus maintaining optimal visualization for the subsequent steps of the procedure (Fig. 3 - Step 6).

Step 3: Perfusion surgery: phlebotomy, exsanguination and fixation

*This section****DOES****require utilization of the dissecting/stereotaxis microscope*.

1. Using the LEICA S9i Digital Stereo Microscope, set the zoom to 0.61x and eyepiece to 10x for a complete magnification of 6.1x. Turn the coarse adjustment knob in the back of the instrument until the heart is centered and in focus. Adjust the overhead lights as needed.
2. Using optometry microsurgical instruments remove pericardial fatty tissue, and thymus from the heart and great vessels (Fig. 4 - Step 1). Alternating between micro grasping forceps and dissecting forceps works well.

**Fig. 4:**
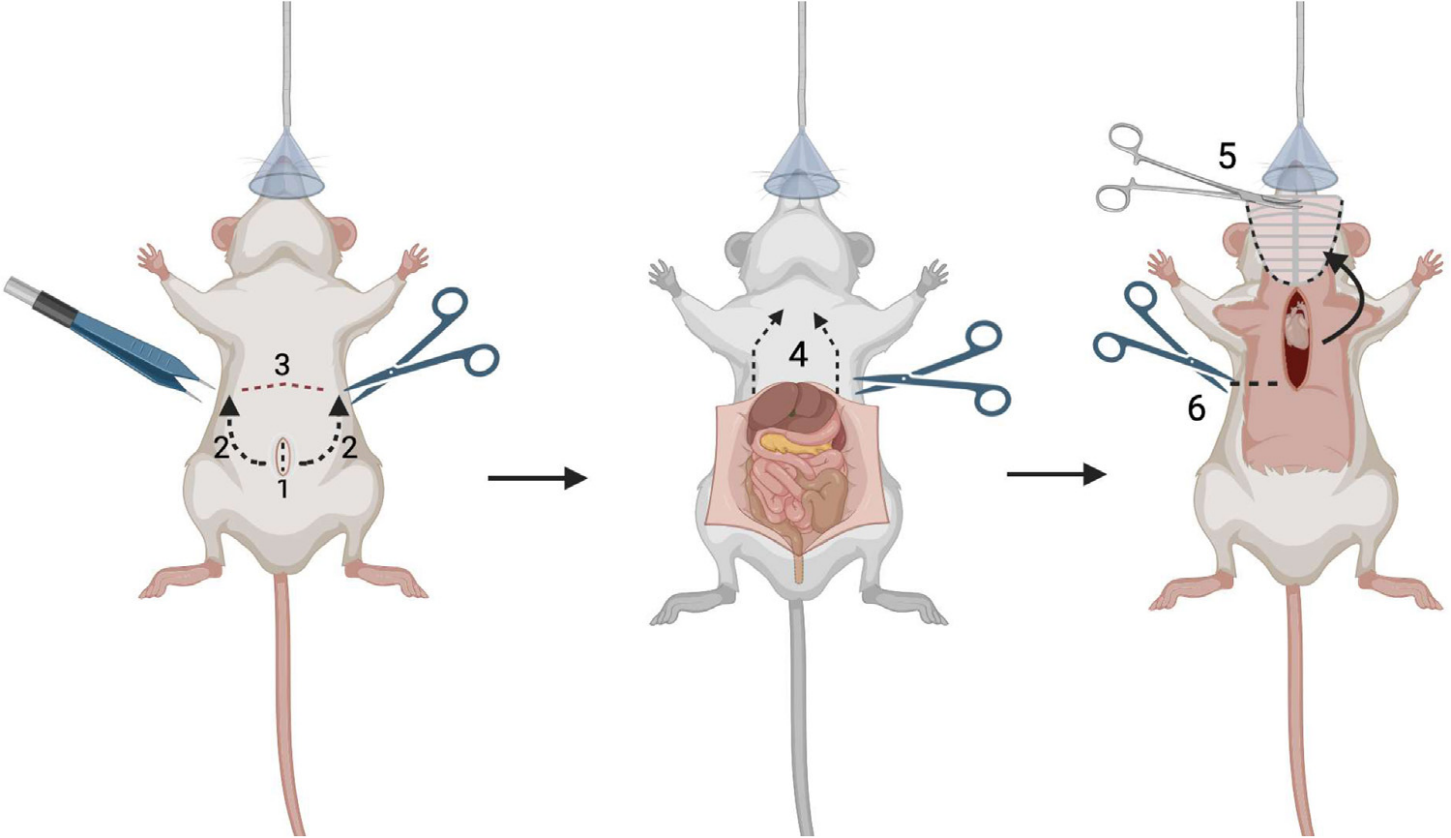
Mouse heart exposure and great vessels visualization.
3. Expose the inferior vena cava (IVC), left and right superior vena cava (SVC), aortic arch, pulmonary artery (PA) and left atrium.
4. Use micro grasping forceps to stabilize the IVC, taking care not to injure the vessel. Insert a 30 G needle attached to 1 mL syringe into the IVC lumen with the tip pointing towards the right atrium.
5. Collect blood, being careful to aspirate slowly without collapsing the vessel (Fig. 4 - Step 3). Rest the back of needle-using-hand on a flat surface for steady blood collection. Aim to collect between 200ul-600uL of blood. Store blood in 1 mL MiniCollect K3 EDTA-coated vacuum-sealed tube on ice. The blood can remain in the K3 EDTA-coated tube on ice until after the cardiectomy is complete (20-30 minutes), the blood can be processed post-tissue processing.
  a. Blood will be centrifuged at 120,000 rpm, at 4°C for 5 minutes.
  b. Pipette supernatant (plasma) and store at -80°C in an Eppendorf tube.
  c. Store red cells and buffy coat at -80°C in a separate Eppendorf tube.

*A 25g mouse requires 4-6mL of buffer to remove blood from the heart*.

6. Using a 25 G needle attached to a 10 mL syringe, load 6 mL of 1x PBS at 4°C.
7. Locate the existing hole in the IVC. Point the needle towards the right atrium within the lumen of the IVC. Steadily inject 3 mL of 1x PBS at 4°C at a rate of 2-3mL/minute (Fig. 4 - Step 4).
8. If done correctly, the pulmonary artery should dilate slightly.
9. Withdraw the needle carefully. Use grasping forceps to stabilize the anterior portion of the left atrium, insert the same needle and inject the remaining 3 mL of 1x PBS at 4°C at a rate of 2-3mL/minute (Fig. 4 - Step 5).
10. If done correctly the aorta should dilate slightly.

*A 25g mouse requires 16-20mL of fixative to cross-link tissues*.

11. Using a 25 G needle attached to a 20 mL syringe, load 20 mL of 4% PFA at 4°C.
12. Locate the existing hole in the IVC. Steadily inject 10 mL of 4% PFA at 4°C at a rate of 2-3mL/minute (Fig. 4 - Step 6).
13. The pulmonary artery will dilate slightly and become translucent/white.
44. Withdraw the needle. Use grasping forceps, steady the anterior portion of the left atrium. Insert the same needle and inject into the existing hole in the left atrium the remaining 10 mL of 4% PFA at 4°C at a rate of 2-3mL/minute (Fig. 4 - Step 7).
15. The aorta will dilate slightly.
16. The aorta, pulmonary artery and both atria should have changed to a dull translucent/white color.

Step 4: Cardiectomy

1. Using micro dissecting scissors and blunt grasping micro forceps, carefully begin cardiectomy.
  a. Start your dissection with the IVC and descending thoracic aorta. The aorta may be located posteriorly to the IVC.
  b. Next, locate each lung and transect the pulmonary artery branches and pulmonary veins away from the right and left lung.
  c. Transect the left and right SVC.
  d. Transect the aortic arch great vessels.
  e. Bluntly dissect away remaining pericardial connective tissue.
  f. Using blunt grasping micro forceps, grasp and elevate the aortic arch to lift the heart vertically. If there is resistance, dissect remaining connective tissue.
  g. Remove heart from mediastinum.

Step 5: Tissue processing

1. Holding the heart from the aorta and pulmonary artery, submerge in 4°C sterile saline (NaCl) for 3-5 minutes to rinse free of residual blood and excess tissue.
2. Once clean, transfer the heart to 5 mL of 4% PFA at 4°C for 18-24 hours, for optimal fixation.
3. Remove heart from 4% PFA solution and wash in ice cold sterile saline (NaCl) for 3-5 minutes.
4. Transfer heart to 5-7 mL of 70% ethanol at room temperature. The heart can remain in this solution until it is ready for paraffin embedding and histological sectioning.

## Method validation

**Fig. 5:**
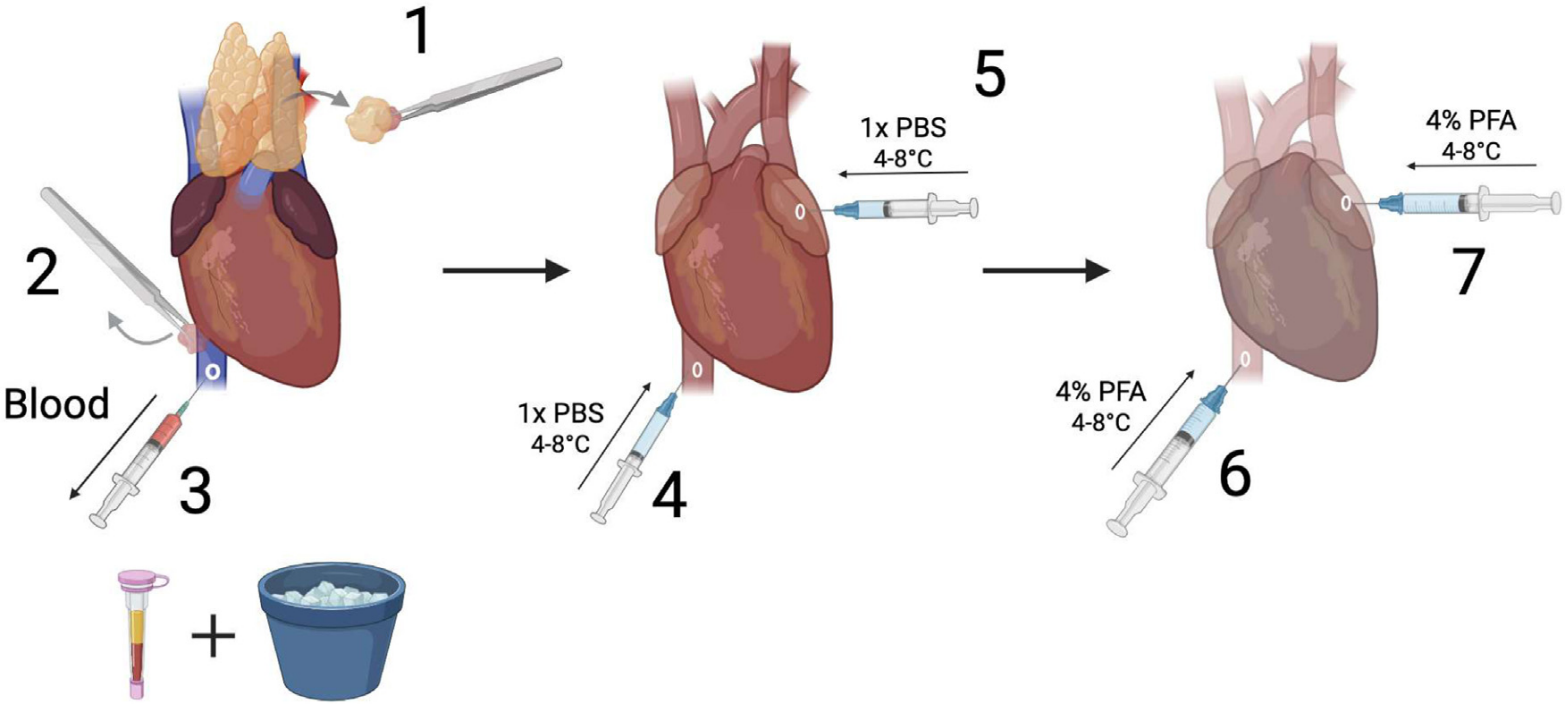
Visualization of great vessels, blood collection, exsanguination and fixation.

**Fig. 6:**
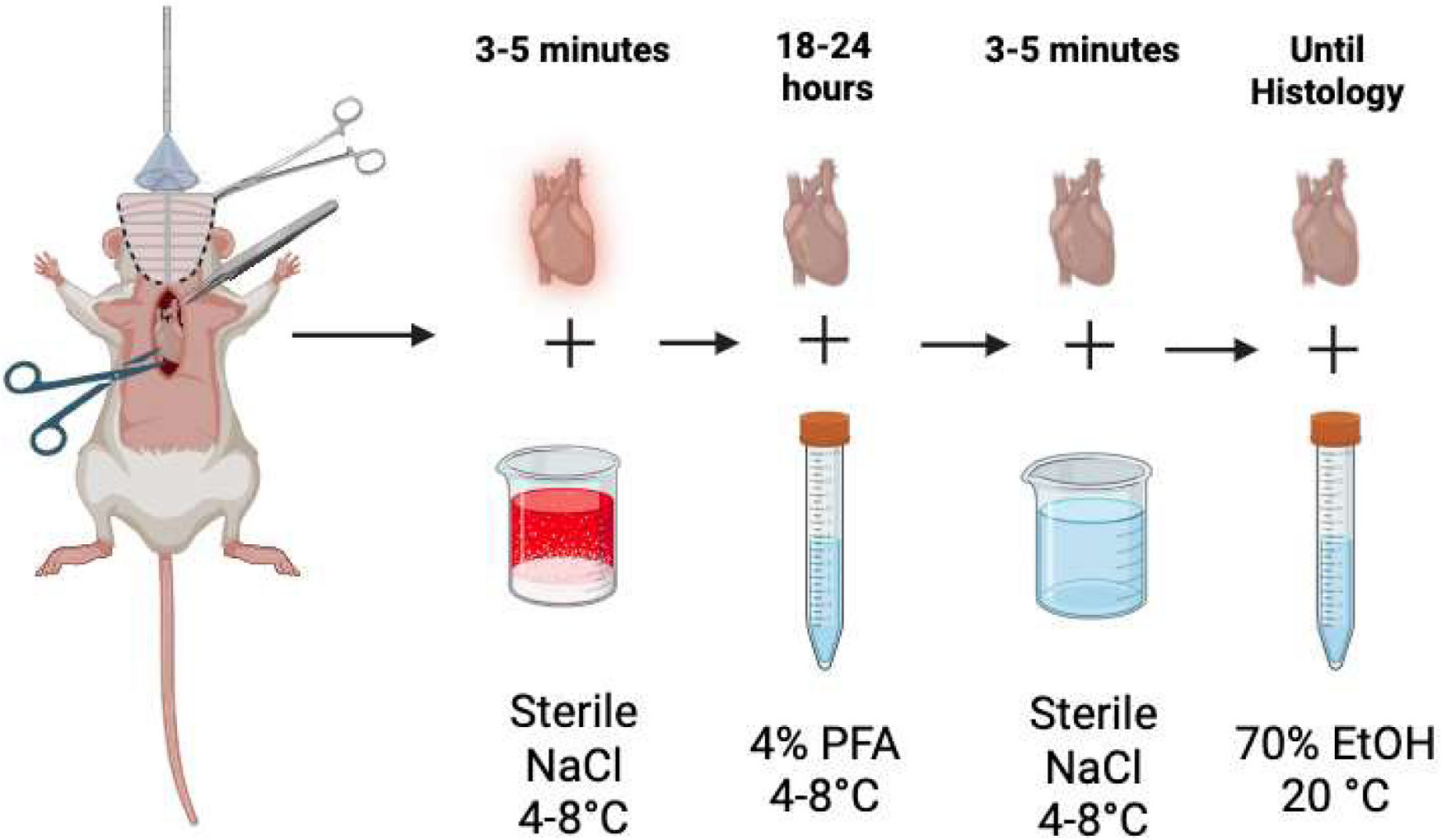
Post-cardiectomy tissue processing scheme prepares the heart for histology. Ensures removal of residual connective tissue, fat, blood and fixative on interior and exterior of tissue. This process is performed with two 100 mL beakers and two 15 mL falcon tubes per heart.

**Fig. 7:**
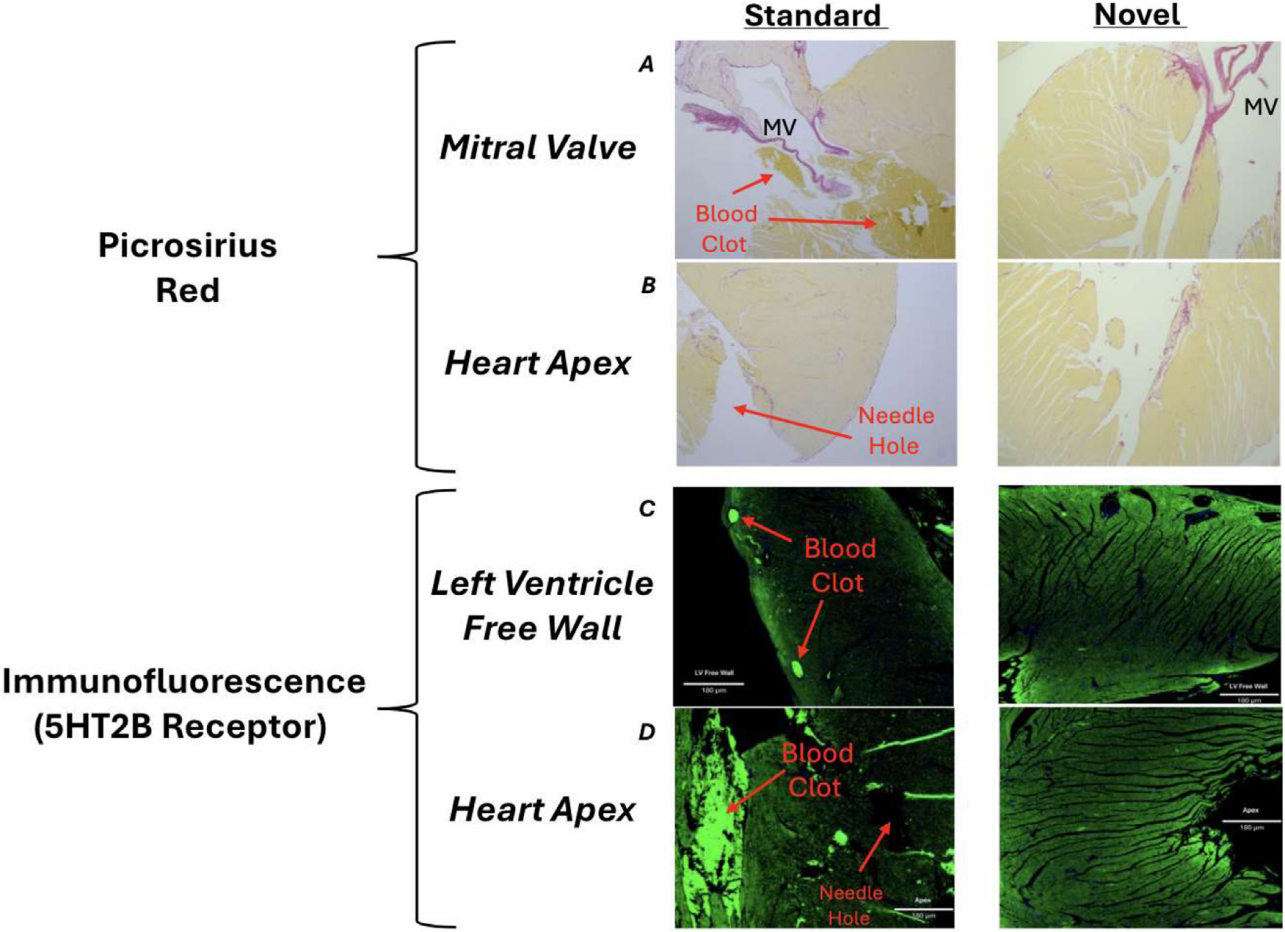
Brightfield Picrosirius red images (A-B) and immunofluorescent images of myocardial Serotonin 2B Receptor (5HTR2B) using Alexa555 secondary antibody, taken by confocal microscopy and processed using NIS-Elements software (C-D). 5 µm Coronal tissue sections of 1-year-old mice myocardium comparing standard protocol to the novel technique. (A) Mitral valve stained red/purple with papillary muscle and interventricular septum stained mustard by Picrosirius red. The standard technique images display large thrombi from poorly evacuated blood products during fixation, with platelets stained yellow near the mitral valve. The novel technique routinely features no blood clots. (B) Heart apex and left ventricle stained by Picrosirius Red. Staining indicates slight collagen arrangement on the border of the left ventricle endocardium. The standard technique displays iatrogenic myocardial trauma to the apical region from the perfusion needle. This causes the fibrosis stain to be discontinuous. (C) Left ventricle free wall immunofluorescence stain by 5HTR2B at 10x magnification. The standard section displays residual thrombi within the coronary vessels represented by bright green circular artifacts in the left ventricle free wall. The novel technique maintains coronary vessels patency. (D) The cardiac apex and left ventricle in the standard section is obscured with blood products represented by numerous bright green clustering artifacts. Additionally, needle trauma is visible as a dark tissue-ripping artifact. The ventricle in the novel technique section is clear and void of myocardial damage.

The purpose of this article is to present an advanced method for the fixation of the whole rodent heart as preparation for histology targeted for researchers interested in characterizing cardiac remodeling and heart valve investigation. This technique allows for a more complete evacuation of blood from the heart chambers and coronary vessels. Additionally, this technique minimizes needle trauma to the myocardium and ensures a thorough distribution of fixative throughout the myocardium. In order to validate this method we compared stained coronal heart sections from the standard perfusion protocol with those from this novel technique (Fig. 5, Fig. 6, Fig. 7).

## Limitations

Not Applicable

## Ethics statements

C57BL/6J male and female mice were purchased from The Jackson Laboratory (Bar Harbor, ME, United States). Additionally, male and female Serotonin Transporter (SERT) KO (Slc6a4 knock-out) mice were purchased from The Jackson Laboratory (Bar Harbor, ME, United States). Mouse sex did not play a significant role in the results of the study. All experiments were reviewed and approved by the Columbia University Institutional Animal Care and Use Committee (IACUC) protocol number AC-AABS7622. This study was conducted in accordance with the National Institutes of Health Guide for the Care and Use of Laboratory Animals (NIH Publication No. 8023; revised 1978).

## CRediT authorship contribution statement

**Cary Karcher:** Conceptualization, Methodology, Validation, Writing – original draft. **Anthony Campbell:** Methodology, Validation, Writing – review & editing. **Dov Levine:** Methodology, Validation, Writing – review & editing. **Morgan Moroi:** Methodology, Validation, Writing – review & editing. **Yaagnik Kosuri:** Methodology, Validation, Writing – review & editing. **Arianna Adamo:** Methodology, Validation, Writing – review & editing. **Giovanni Ferrari:** Conceptualization, Supervision, Writing – review & editing.

## Declaration of competing interest

The authors declare that they have no known competing financial interests or personal relationships that could have appeared to influence the work reported in this paper.

## Data Availability

Data will be made available on request.
